# Predicting the occurrence of major adverse cardiac events within 30 days of a vascular surgery: an empirical comparison of the minimum p value method and ROC curve approach using individual patient data meta-analysis

**DOI:** 10.1186/s40064-016-1936-8

**Published:** 2016-03-09

**Authors:** Thuva Vanniyasingam, Reitze N. Rodseth, Giovanna A. Lurati Buse, Daniel Bolliger, Christoph S. Burkhart, Brian H. Cuthbertson, Simon C. Gibson, Elisabeth Mahla, David W. Leibowitz, Bruce M. Biccard, Lehana Thabane

**Affiliations:** Department of Clinical Epidemiology and Biostatistics, McMaster University, Health Sciences Centre, Room 2C7, 1280 Main Street West, Hamilton, ON L8S 4K1 Canada; Perioperative Research Unit, Department of Anaesthetics, Nelson R. Mandela School of Medicine, University of KwaZulu-Natal, Pietermaritzburg, South Africa; Department of Anaesthetics, Grey’s Hospital, Pietermaritzburg, South Africa; Department of Anaesthesia, Surgical Intensive Care, Prehospital Emergency Medicine and Pain Therapy, University Hospital Basel, Basel, Switzerland; Department of Anaesthesiology, Kantonsspital Graubunden, Chur, Switzerland; Department of Critical Care Medicine, Sunnybrook Health Sciences Centre and University of Toronto, Toronto, ON Canada; Queen Elizabeth University Hospital, Glasgow, UK; Department of Anesthesiology and Intensive Care Medicine, Medical University of Graz, Graz, Austria; Division of Cardiology, Hadassah-Hebrew University Medical Center, Jerusalem, Israel; Department of Anaesthesia and Perioperative Medicine, University of Cape Town, Cape Town, South Africa; Population Health Research Institute, Hamilton Health Sciences, Hamilton, ON Canada; Biostatistics Unit, St Joseph’s Healthcare, Hamilton, ON Canada; Departments of Paediatrics and Anaesthesia, McMaster University, Hamilton, ON Canada; Centre for Evaluation of Medicine, St Joseph’s Healthcare, Hamilton, ON Canada

**Keywords:** Vascular surgery, Minimum p value, ROC curve approach, Pre-operative risk, Biostatistics, Cardiovascular epidemiology

## Abstract

We aimed to compare the minimum p value method and the area under the receiver operating characteristics (ROC) curve approach to categorize continuous biomarkers for the prediction of postoperative 30-day major adverse cardiac events in noncardiac vascular surgery patients. Individual-patient data from six cohorts reporting B-type natriuretic peptide (BNP) or N-terminal pro-B-type natriuretic peptide (NTproBNP) were obtained. These biomarkers were dichotomized using the minimum p value method and compared with previously reported ROC curve-derived thresholds using logistic regression analysis. A final prediction model was developed, internally validated, and assessed for its sensitivity to clustering effects. Finally, a preoperative risk score system was proposed. Thresholds identified by the minimum p value method and ROC curve approach were 115.57 pg/ml (p < 0.001) and 116 pg/ml for BNP, and 241.7 pg/ml (p = 0.001) and 277.5 pg/ml for NTproBNP, respectively. The minimum p value thresholds were slightly stronger predictors based on our logistic regression analysis. The final model included a composite predictor of the minimum p value method’s BNP and NTproBNP thresholds [odds ratio (OR) = 8.5, p < 0.001], surgery type (OR = 2.5, p = 0.002), and diabetes (OR = 2.1, p = 0.015). Preoperative risks using the scoring system ranged from 2 to 49 %. The minimum p value method and ROC curve approach identify similar optimal thresholds. We propose to replace the revised cardiac risk index with our risk score system for individual-specific preoperative risk stratification after noncardiac nonvascular surgery.

## Background

There are over 200 million individuals receiving major noncardiac surgery worldwide annually (Ford et al. 2010), many carrying a great risk of major adverse cardiovascular events (MACE), including myocardial infarction (MI) and mortality (Rodseth et al. 2011). Preoperative risk-stratification to identify high-risk patients is used to improve perioperative management. The revised cardiac risk index (RCRI) (Lee et al. 1999; Boersma et al. 2005; Fleisher et al. 2007; Poldermans et al. 2009) is the leading instrument used, however it was derived from a heterogeneous noncardiac population, and is unable to produce individual-specific risk scores (Biccard and Rodseth 2011). A systematic review of the RCRI found that prediction of cardiac events (cardiac death, MI, and nonfatal cardiac arrest) are notably less accurate for noncardiac vascular surgery patients (Ford et al. 2010).

Two hormones, B-type natriuretic peptide (BNP) and N-terminal pro-B-type natriuretic peptide (NTproBNP) have recently been investigated as prognostic biomarkers (Rajagopalan et al. 2008, 2011; Rodseth et al. 2008, 2011). They are released into the blood by ventricular cardiomyocytes when there is mechanical or ischemic strain on the atrial or ventricular wall (Rodseth et al. 2011; Harrison et al. 2002). Elevation in either of these hormone concentrations increase the risk of 30-day MACE (Rodseth et al. 2011; Karthikeyan et al. 2009). Optimal thresholds are under investigation.

Rodseth et al. conducted an individual patient data meta-analysis and proposed thresholds of 116 pg/ml for BNP and 277.5 pg/ml for NTproBNP for predicting 30-day MACE (Rodseth et al. 2011). These values were identified using the receiver operating characteristic (ROC) curve approach for categorizing continuous variables (Cook 2008; Zhu et al. 2010). There are other less commonly used discrimination methods, such as the minimum p value method, which may identify a more predictive threshold. Comparisons between these methods have yet to be investigated.

We aimed to (1) explore the BNP and NTproBNP thresholds from Rodseth et al., (2) incorporate the best performing discrimination method into a final predictive model for 30-day MACE after a noncardiac vascular surgery, and (3) propose a decision tool for clinicians to determine individual-patient risk. The secondary objectives were to explore individual prediction rules for all-cause mortality, cardiac death, and nonfatal MI within 30 days after a vascular surgery.

## Results

A total of 850 participants who received noncardiac nonvascular surgery were included in our dataset. Only 75 of these individuals experienced a MACE within 30 days (9 %). Participants had an average age of 65.4 years (sd = 12.1), and approximately half were males (391/850, 46 %). Seventy-five patients experienced MACE within 30 days while the modified RCRI used by Rodseth et al. predicted 54 (72 %) of these events. Of those who experienced a MACE, 56 % had a history of coronary artery disease (42/75), 25 % had a history of diabetes mellitus (25/75), 14 % had a history of congestive heart failure (14/75), and 8 % had a history of cerebrovascular disease (8/75). The type of surgery for four participants was not specified and these individuals were not included in our univariate and multivariable analyses. None experienced a MACE within 30 days. Patient characteristics are presented in Table 1 and additional participant information can be found in Rodseth et al.’s study (Rodseth et al. 2011).

**Table 1:** Participant characteristics

Variable	Total (n = 850)	MACE30 (n = 75)	NO MACE30 (n = 775)	Test statistic	p
Age^a^ (yr): mean (SD)	65.4 (12.1)	69.4 (8.8)	65.0 (12.3)	3.07	0.002
Sex (men): n (%)	391 (46.0)	36 (48.0)	355 (45.8)	0.15	0.696
+Missing	218 (26.0)				
Type of vascular surgery: n (%)				2.54	0.110
(a) Infrainguinal	629 (74.0)	50 (66.7)	579 (74.7)		
(b) Aortoiliac	217 (25.5)	25 (33.3)	192 (24.8)		
(c) Not specified^b^	4 (0.5)	0	4 (0.5)		
RCRI class: n (%)				12.50	0.002
(a) Low (RCRI 0)	320 (37.6)	19 (25.3)	301 (38.8)		
(b) Intermediate (RCRI 1 or 2)	476 (56.0)	45 (60.0)	431 (55.6)		
(c) High (RCRI 3)	54 (6.4)	11 (14.7)	43 (5.5)		
RCRI components: n (%)					
Coronary artery disease	327 (38.5)	42 (56.0)	285 (36.8)	10.68	0.001
Congestive heart failure	64 (7.5)	14 (18.7)	50 (6.5)	14.65	<0.001
Cerebrovascular disease	145 (17.1)	8 (10.7)	137 (17.7)	2.38	0.123
Diabetes mellitus	204 (24.0)	25 (33.3)	179 (23.1)	3.93	0.048
Creatinine (≥2 mg/dl)	28 (3.3)	6 (8.0)	22 (2.8)	5.72	0.017
BNP: median (min, max)		132 (0, 3893)	12 (0, 3139)	6.54^c^	<0.001^c^
NTproBNP: median (min, max)		534 (24, 25,780)	204 (5, 6172)	3.02^c^	0.003^c^

*yr* years, *SD* standard deviation, *P. Chi* Pearson Chi square test, *RCRI* revised cardiac risk index, *BNP* B-type natriuretic peptide, *NTproBNP* N-terminal pro-B-type natriuretic peptide

^a^Analysis performed using t test

^b^Level not included in analysis

^c^Analysis performed with Mann-U-Whitney test; other variables were analyzed with Chi squared tests

### Minimal p value method for obtaining thresholds

**Fig. 1:**
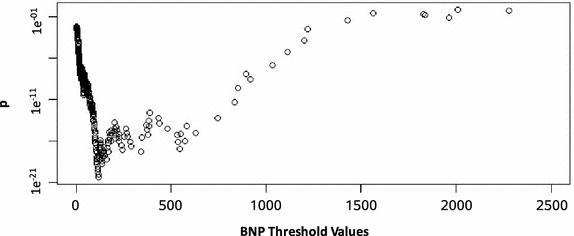
A minimum p value analysis demonstrating potential BNP threshold values and corresponding p values. This *graph* presents the corresponding p value of each Chi square test performed on a series of potential B-type natriuretic peptide (BNP) thresholds to predict 30-day MACE after vascular surgery. The threshold with the smallest p value is set as the optimal threshold for dichotomizing NTproBNP

**Fig. 2:**
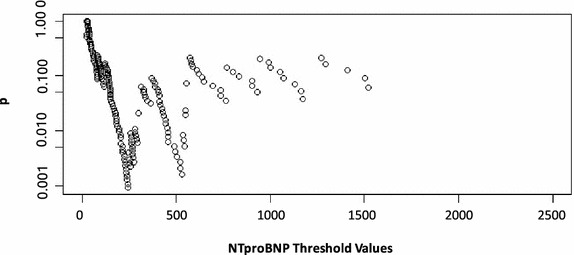
A minimum p value analysis demonstrating potential BNP threshold values and corresponding p values. This *graph* presents the corresponding p value of each Chi square test performed on a series of potential NTproBNP thresholds to predict 30-day MACE after vascular surgery. The threshold with the smallest p value is set as the optimal threshold for dichotomizing NTproBNP

We excluded 0 pg/ml as a threshold and removed 5 % of outliers from BNP and NTproBNP. The remaining 303 BNP thresholds (range 2–2322 pg/ml) and 204 NTproBNP thresholds (range 22–1572 pg/ml) were investigated. The thresholds corresponding to the minimal p values for BNP and NTproBNP were 115.57 pg/ml (p < 0.001, *χ*^2^ = 88.79, df = 1, RR = 2.09) and 241.70 pg/ml (p = 0.001, *χ*^2^ = 10.98, df = 1, RR = 3.33), respectively (Figs. 1 and 2). These were very close to Rodseth et al.’s ROC thresholds (BNP: 116 pg/ml, NTproBNP: 277.5 pg/ml). Our p value adjustment optimal thresholds were statistically significant with MACE (*p*_*BNP*_ < 0.001, *p*_*NTproBNP*_ < 0.001). Rodseth’s ROC curve results for BNP and NTproBNP were 116 pg/ml (66 % sensitivity, 82 % specificity) and 277.5 pg/ml, respectively (Rodseth et al. 2011). The final BNP and NTproBNP thresholds were combined to form our minimum p value method (MPM) threshold variable.

### Comparing logistic regression methods

The comparison between the previously reported model (ROC threshold, surgery type, and diabetes mellitus) with our model MPM threshold, surgery type, diabetes mellitus) are presented in Table 2. The MPM threshold had a slightly higher odds ratio and a smaller p value than the ROC threshold, indicating a stronger association with 30-day MACE. The new model had a larger AUC and a smaller AIC, indicating a better-fit. We used MPM threshold due to its slightly better overall performance.

**Table 2:** Comparison of ROC and MPM thresholds using logistic regression analysis

Models and predictors	OR (95 % CI)	p	AUC	AIC
ROC threshold				
BNP ≥ 116 pg/ml or NTproBNP ≥ 277.5 pg/ml;	8.4 (4.98,14.11)	<0.001	0.768	431.63
Ref = BNP < 116 or NTproBNP < 277.5 pg/ml				
Surgery type				
Aortoiliac; Ref = Infrainguinal surgery	2.4 (1.33, 4.37)	0.004		
Diabetes				
Yes; Ref = No	2.0 (1.10, 3.54)	0.023		
MPM threshold				
BNP ≥ 115.57 pg/ml or NTproBNP ≥ 246.7 pg/ml;	8.5 (5.03, 14.41)	<0.001	0.777	430.16
Ref = BNP < 115.57 pg/ml or NTproBNP < 246.7 pg/ml				
Surgery type				
Aortoiliac; Ref = Infrainguinal surgery	2.5 (1.40, 4.60)	0.002		
Diabetes				
Yes; Ref = No	2.1 (1.15, 3.71)	0.015		

ROC threshold is an indicator variable of BNP and NTproBNP thresholds determined by the ROC curve method (Allison 2012); MPM threshold is an indicator variable of BNP and NTproBNP thresholds determined by the minimum p value method

*Ref* reference level, *OR* odds ratio, *CI* confidence interval, *AUC* area under the ROC curve

### Development of a final prediction model

We first evaluated the impact of each predictor in Rodseth et al.’s model by individually and then collectively adding diabetes and surgery type to a model with MPM threshold. A model with only MPM threshold yielded a p_MPM threshold_ < 0.001 and an AUC of 0.72. Diabetes was not significant when adjusting for MPM threshold (p_diabetes_ = 0.100, AUC = 0.74) while surgery type was (p_surgerytype_ = 0.013, AUC = 0.76). In the full model, however, surgery type and diabetes were significant (Table 2).

We further investigated the MPM threshold with other significant predictors (p < 0.05) from our univariate analyses (Table 1): age greater than 65 years, history of coronary artery disease history, congestive cardiac failure history, diabetes mellitus, and renal insufficiency. We had a low number of observations for study 5 (n_5_ = 3), therefore we excluded these levels from our logistic regression analysis. The following predictors were significantly associated with 30-day MACE and were included in our final model: MPM threshold (OR = 8.5, 95 % CI 5.03–14.41, p < 0.001), surgery type (OR 2.5, 95 % CI 1.40–4.60, p = 0.002), and diabetes (OR 2.1, 95 % CI 1.11–3.84, p = 0.023). The variance inflation factors were all less than 1.2 and the tolerance levels for each explanatory variable were greater than or equal to 0.9, suggesting low multicollinearity among predictors. The final model is presented as the development model in Table 3, which also presents the results from our sensitivity and validation analysis.

**Table 3:** Determining a prediction model for 30-day MACE

Models and predictors	OR (95 % CI)	p	AUC
*Development model*			
MPM threshold			
BNP ≥ 115.57 pg/ml or NTproBNP ≥ 241.7 pg/ml;	8.5 (5.03, 14.41)	<0.001	0.777
Ref = BNP < 115.57 pg/ml or NTproBNP < 241.7 pg/ml			
Surgery type			
Aortoiliac; Ref = infrainguinal surgery	2.5 (1.40, 4.60)	0.002	
Diabetes			
Yes; Ref = No	2.1 (1.15, 3.71)	0.015	
*Internal validation*			
MPM threshold			
BNP ≥ 115.57 pg/ml or NTproBNP ≥ 241.7 pg/ml;	8.6 (4.95, 14.65)	<0.001	0.793
Ref = BNP < 115.57 pg/ml or NTproBNP < 241.7 pg/ml			
Surgery type			
Aortoiliac; Ref = infrainguinal surgery	2.6 (1.37, 4.68)	0.004	
Diabetes			
Yes; Ref = No	2.1 (1.11, 3.84)	0.023	
*Sensitivity analysis*			
Mixed effects logistic regression model			
MPM threshold			
BNP ≥ 115.57 pg/ml or NTproBNP ≥ 241.7 pg/ml;	10 (5.59, 18.06)	<0.001	0.776
Ref = BNP < 115.57 pg/ml or NTproBNP < 241.7 pg/ml			
Surgery type			
Aortoiliac; Ref = infrainguinal surgery	2.8 (1.51, 5.31)	0.001	
Diabetes			
Yes; Ref = No	1.6 (0.83, 2.96)	0.17	
Generalized estimating equation			
MPM threshold			
BNP ≥ 115.57 pg/ml or NTproBNP ≥ 241.7 pg/ml;	9.4 (3.81, 23.33)	<0.001	N/A
Ref = BNP < 115.57 pg/ml or NTproBNP < 241.7 pg/ml			
Surgery type			
Aortoiliac; Ref = infrainguinal surgery	2.7 (1.27, 5.73)	0.01	
Diabetes			
Yes; Ref = No	1.8 (0.95, 3.46)	0.072	

ROC threshold is an indicator variable of BNP and NTproBNP thresholds determined by the ROC curve method (Allison 2012); MPM threshold is an indicator variable of BNP and NTproBNP thresholds determined by the minimum p value method

*Ref* reference level, *OR* odds ratio, *CI* confidence interval, *AUC* area under the ROC curve

### Validation and sensitivity analysis

**Fig. 3:**
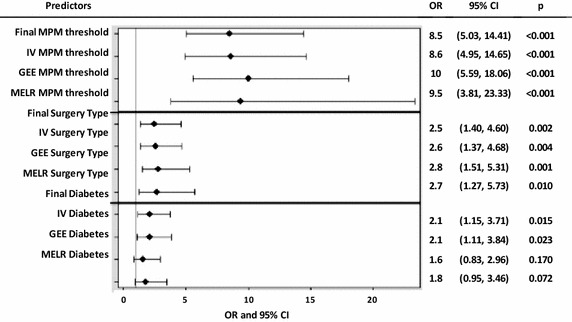
Forest plot of final model, internal validation model, and sensitivity models. Four models were created: (1) a final prediction model, (2) internal validation (IV) model, generalized estimating equations (GEE), and (3) mixed effects logistic regression (MELR) model with predictors MPM threshold, surgery type, and history of diabetes mellitus. This plot presents the odds ratio (OR), 95 % confidence interval (CI) and p value for each predictor of each model. MPM threshold is a composite predictor of BNP and NTproBNP thresholds determined by the minimum p value method [MPM threshold = 0 (reference) if BNP < 115.57 pg/ml or NTproBNP < 241.7 pg/ml; MPM threshold = 1 if BNP > 115.57 pg/ml or NTproBNP > 241.7 pg/ml). Surgery type is the type of noncardiac vascular surgery [infrainguinal (reference) vs. aortoiliac]. Diabetes is an indicator of whether or not an individual was diagnosed with diabetes [no (reference) vs. yes]

Our internal validation model identified similar estimates as our final model (MPM threshold: OR 8.6, 95 % CI 4.95–14.65, p < 0.001; surgery type: OR 2.6, 95 % CI 1.37–4.68, p = 0.004; diabetes: OR 2.1, 95 % CI 1.11–3.84, p = 0.023; AUC = 0.79). Diabetes was not a significant predictor in our mixed effects logistic regression model and our GEE (p > 0.05, α = 0.05). This indicates that some heterogeneity existed among the data. Figure 3 presents the results of each model, separated by predictor to clearly depict the variability between each model.

### Producing a points system

We created a scoring system by first assigning a 0 point to each predictor’s reference level (the least unhealthy level). *B*, the number of regression units needed for one point in the scoring system and also the smallest regression coefficient in the model, was 0.7262 (*β*_*minimum*_ = *β*_*diabetes*_ = 0.7262). This was used to divide and round all remaining regression coefficients to obtain their corresponding points. History of diabetes was assigned a point of 1, BNP/NTproBNP concentration values greater than the MPM threshold a point of 3, and aortoiliac surgery a point of 1. Thus, individuals scheduled for noncardiac vascular surgery would receive a total score ranging from 0 to 5, which corresponds to an associated risk ranging from 2.49 to 49.10 % (Table 4).

**Table 4:** Scoring system for 30-day preoperative risk

Point total	Estimate of risk (%)
0	2.49
1	5.01
2	9.83
3	18.40
4	31.80
5	49.10

The point totals are determined by adding points for each level of the predictors MPM threshold, surgery type, and diabetes that matches each patient’s information. Percentages accumulate to more than 100 % due to rounding during point creation

### Secondary analyses

We produced mixed effect logistic regression models for our secondary outcomes (all-cause mortality, cardiac death, and non-fatal MI) to account for the within or between study heterogeneity observed in our sensitivity analysis (Table 5). Diabetes was not significant and was removed from all three models. The MPM threshold remained a statistically significant prognostic factor for each outcome. Surgery type was not significantly associated with cardiac death. The AUCs of each model are greater than 0.7, demonstrating a moderate fit for each outcome with only MPM threshold and surgery type as predictors.

**Table 5:** Prediction models for all-cause mortality, cardiac death, and non-fatal myocardial infarction

Models and predictors	OR (95 % CI)	p	AUC
Outcome = all-cause mortality			
MPM threshold			
BNP ≥ 115.57 pg/ml or NTproBNP ≥ 241.7 pg/ml;	6.7 (2.76, 16.10)	<0.001	0.714
Ref = BNP < 115.57 pg/ml or NTproBNP < 241.7 pg/ml			
Surgery type			
Aortoiliac; Ref = infrainguinal surgery	2.8 (1.16,6.79)	0.022	
Outcome = cardiac death			
MPM threshold			
BNP ≥ 115.57 pg/ml or NTproBNP ≥ 241.7 pg/ml;	9.2 (3.10, 27.37)	<0.001	0.750
Ref = BNP < 115.57 pg/ml or NTproBNP < 241.7 pg/ml			
Surgery type			
Aortoiliac; Ref = infrainguinal surgery	2.6 (0.91,7.64)	0.075	
Outcome = nonfatal MI			
MPM threshold			
BNP > 115.57 pg/ml or NTproBNP > 241.7 pg/ml;	8.7 (4.60, 16.33)	<0.001	0.787
Ref = BNP < 115.57 pg/ml or NTproBNP < 241.7 pg/ml			
Surgery type			
Aortoiliac; Ref = infrainguinal surgery	2.1 (1.06,4.17)	0.034	

Three models for each secondary outcome are presented. MPM threshold is an indicator variable of BNP and NTproBNP optimal thresholds determined by the minimum p value method

*Ref* reference level, *OR* odds ratio, *CI* confidence interval, *AUC* area under the ROC curve

## Discussion

We used data from six cohort studies (n = 832 participants) to propose an individual-specific risk prediction scoring system for 30-day MACE after noncardiac vascular surgery, based on a combined threshold factor for BNP and NTproBNP concentration levels, surgery type, and history of diabetes mellitus. The points system derived from this prediction model can allow clinicians to determine patients’ risk, ranging from 2.49 to 49.10 %, in real time. Thresholds for BNP and NTproBNP were derived using the minimum p value method, which had a slightly better performance than the ROC curve thresholds in our prediction model.

There are various methods for categorizing continuous variables (Williams et al. 2006). Some use clinically relevant thresholds which are arbitrary, and may not be representative of a predictor’s true prognostic capability. Others use data-dependent approaches such as splitting by the mean or by percentiles (25th, median, 75th). Outcome-based methods, such as the ROC curve approach and the minimum p value method, systematically identify an “optimal” threshold from a set of values, using statistical methods and clinical guidance. Previous studies have derived ROC thresholds and validated them using different approaches such as bootstrapping or leave one-out cross-validation (Nougaret et al. 2015). To our knowledge, no study has compared the ROC curve method to the minimum p value method to determine which will identify more informative thresholds. The BNP and NTproBNP thresholds themselves differ by 126.13 pg/ml (115.57 and 241.70 pg/ml, respectively) due to their composition. The half-life of NTproBNP (120 min) is six times longer than that of BNP (20 mins), making NTproBNP concentration levels higher than BNP values (Weber and Hamm 2006).

In our analysis of other predictors, surgery type was clinically important and was included in the final prediction model of 30-day MACE. It was not significant in the univariate analysis (p = 0.229), however it was significant in our multivariable model. In contrast, congestive heart failure was significant (p < 0.001) in our univariate analysis, however was excluded in the final model. Despite a small VIF (0.98), literature describes a strong association between this factor and BNP/NTproBNP concentration levels, thus to avoid potential collinearity it was excluded (Harrison et al. 2002; Maisel 2002). Our secondary analyses identified MPM threshold and surgery type as significant predictors for our secondary outcomes of all-cause mortality and non-fatal MI. For our cardiac death outcome, only MPM threshold was statistically significant. Further exploration of prognostic factors is needed to see the impact of MPM threshold on each outcome and to determine optimal prediction rules.

This study has three key strengths. First, we compared the performance of methods that are widely used for categorizing continuous predictors. Second, we present an internally-valid prediction model for 30-day MACE based on easily attainable measures and robust thresholds. Third, we describe an easy method to convert regression coefficients into risk-prediction scores, which simplifies preoperative risk classification for clinicians.

This study had several limitations. First, we used a composite predictor for BNP and NTproBNP, since no study reported both measures. NTproBNP has a more stable composition with possibly less sensitivity to sudden haemodynamic shifts, making it a potentially better biomarker than BNP for adverse outcomes (Rodseth et al. 2008; Rodseth 2009). However, limited data on NTproBNP was available (n = 218 participants), and the differing optimal thresholds derived using the minimum p value method and the ROC curve approach suggested that they were less robust than the BNP thresholds. Second, the minimum p value method is subject to inflation of Type I error rates (Faraggi and Simon 1996) however we adjusted for this using three formulae to reassess our p values. Third, in our regression analysis, we excluded the ‘not specified’ level of surgery type while Rodseth et al.’s included it (Rodseth et al. 2011). This may explain the slight discrepancy between our ROC threshold variable and their’s. We used different statistical software to run our analyses, and were unable to check the impact of excluding this level.

Future research should further investigate NTproBNP to finalize its optimal threshold, and to determine whether it outperforms BNP in risk prediction. Furthermore, a twofold cross-validation technique could be used to identify optimal thresholds with relatively accurate type I error rates and unbiased estimates of effect size (Maisel 2002; Faraggi and Simon 1996), however larger datasets are required (Faraggi and Simon 1996; Mazumdar et al. 2003). Also, comparing reclassification of patients using the RCRI and using our final prediction model would confirm which approach was more appropriate for pre-operative risk prediction among vascular surgery patients. Lastly, our proposed prediction score should be investigated in the clinical setting to evaluate its impact on patient outcomes and overall perioperative management.

## Conclusions

The minimum p value method presents similar optimal thresholds as the ROC curve approach in prediction of 30-day MACE after noncardiac vascular surgery. We propose to replace the RCRI with our risk score system. Reclassification of patients using the RCRI and our final prediction model, along with further investigation of NTproBNP, should be performed to validate our thresholds and determine the most optimal model for pre-operative risk prediction among vascular surgery patients.

## Methods

### Study population

We used data from a previously conducted individual patient data meta-analysis (Rodseth et al. 2011). Briefly, six cohort studies on patients receiving elective noncardiac vascular surgery (n = 850) investigated associations between preoperative BNP/NTproBNP concentration levels and postoperative cardiovascular outcomes within 30-days, including all-cause mortality, cardiac death, non-fatal MI, and MACE (composite of all-cause mortality and non-fatal MI). Five studies reported BNP (n = 632 participants) (Bolliger et al. 2009; Biccard and Naidoo 2011; Cuthbertson et al. 2007; Gibson et al. 2007; Leibowitz et al. 2008), and one reported NTproBNP (n = 218 participants) (Mahla et al. 2007) concentration levels. No study reported both measurements. Participant characteristics were reported as mean and standard deviation (sd) for continuous variables, and counts and percentages for categorical variables. BNP and NTproBNP are skewed continuous variables and were reported using medians, minimum and maximum values.

### Comparison of methods

#### ROC curve approach

The ROC curve approach is used to measure the accuracy of a test in determining a dichotomous outcome (Maisel 2002). This approach determines an optimal threshold that has the highest accuracy for the prediction of a dichotomous outcome. A graphical presentation of an ROC curve reveals 1-specificity values (false positive rate, x-axis) and the sensitivity values (true positive rate, y-axis) for each potential threshold. Previously, optimal thresholds of 116 pg/ml for BNP (66 % sensitivity, 82 % specificity) and 277.5 pg/ml for NTproBNP (unspecified sensitivity/specificity) were identified. Since no individual reported both BNP and NTproBNP measures, these thresholds were combined into one indicator variable, hereafter referred to as the ROC threshold. Investigation of other prognostic factors revealed a final logistic regression model with ROC threshold, type of surgery, and diabetes mellitus as significant prognostic factors of 30-day MACE. This model was reproduced to make appropriate comparisons between the ROC curve approach and the minimum p value method.

#### Minimum p value method

The minimum p value method is another method to determine critical thresholds of dichotomous outcomes. A portion of extreme values are removed and each remaining observation is assessed as a potential threshold (Harrison et al. 2002). Chi squared tests were used to test the significant difference between the two groups (low vs. high risk) for 30-day MACE at each threshold. The threshold with the smallest p value is identified as the optimal cut-point where individuals with elevated levels beyond the threshold are considered to have a high risk of 30-day MACE. The Chi square statistics (χ^2^), corresponding p values (p), degrees of freedom (df), and relative risk (RR) measures are reported for the final BNP and NTproBNP thresholds.

Inflation of Type 1 error caused by multiple-testing can arise and inflate the p values. We evaluated three formulae designed to reduce type I error inflation by adjusting the p values for each of the final BNP and NTproBNP thresholds (Mazumdar and Glassman 2000). The smallest p value between two of these formulae (Miller and Siegmund’s 1982; Lausen and Schumaker’s 1996) was used to determine if each threshold was statistically significant (p < 0.05). These two final cut points were combined into one indicator variable, hereafter referred to as the minimum p value method threshold (MPM threshold).

#### Comparing methods

We compared the odds ratios (ORs) and corresponding p values of BNP and NTproBNP thresholds using two logistic regression models, the previously reported model (ROC threshold, surgery type, and diabetes mellitus) and a new model (MPM threshold, surgery type, diabetes mellitus). A stronger association of the MPM or ROC threshold with 30-day MACE was implied by a higher OR and a smaller p value. Goodness-of-fit statistics for both models were compared, where a better-fit model had a higher area under the ROC curve (AUC) and lower Akaike information criteria (AIC). We used RStudioVersion 0.96.316 software (RStudio 2012) for the minimum p value method. All other analyses were performed in Version 9.3 of SAS (Cary, NC).

### Determining a prediction model for 30-day MACE

#### Development of a final prediction model

The primary predictor was the optimal threshold variable (MPM or ROC). Other prognostic factors of 30-day MACE were type of surgery (infra-inguinal or aorto-iliac); patient age; and history of diabetes mellitus, congestive cardiac failure, coronary artery disease, cerebrovascular disease, and renal failure (creatinine >2 mg/dl). These were evaluated using t-tests or Chi square tests for continuous or categorical variables, respectively. Significant predictors (p < 0.05) were added to the model using a hierarchical approach. Final prognostic factors were assessed for multicollinearity with the variance inflation factor (VIF), where a VIF greater than 10 indicated high collinearity (Allison 2012).

#### Internal validation via bootstrapping

We produced logistic regression models for 1000 bootstrapped samples, sampled with replacement, from our dataset (Fox 2002). We averaged these models to produce a final validation model to compare the ORs, 95 % CIs, p values, and the AIC with our prediction model.

#### Sensitivity analysis

To test whether our final model was sensitive to potential clustering effects created from our multi-leveled data structure, we compared our estimates with a mixed effects logistic regression model and generalized estimating equations (GEE). Our mixed effects logistic regression model was comprised of fixed effects (the covariates) and one random effect (study). The error and random effect term are assumed to follow a normal distribution with a mean of zero (Heo and Leon 2005). Our GEE assumed an exchangeable correlation matrix to consider a constant correlation between responses within a study and the same correlation structure was assumed across studies (Neuhaus et al. 1991).

### Producing a points system for preoperative risk of 30-day MACE

We produced a points system based on our final model. The reference level of each variable from our regression analysis was the least unhealthy state, and assigned a value of 0 (*W*_*iREF*_). Each additional level outside the reference level was given a positive value of (e.g. 1, 2). These levels were referred to as *W*_*ij*_, for the *j*th level of the *i*th predictor variable. We then calculated points for each level using:

$$Points_{ij} = \frac{{\hat{\beta }_{i} \left( {W_{ij} - W_{iREF} } \right)}}{B},$$

where, $\hat{\beta }_{i} ,$ is the parameter estimate for the *i*th predictor variable and *B* is the smallest parameter estimate (*β*_*min*_) among all of the regression coefficients in the model. *B* represents the number of regression units needed for one point in the scoring system. Using an approximation of the logistic regression formula, we created point totals (rounded to the nearest whole number) and corresponding risk estimates for all possible patient profiles (Sullivan et al. 2004).

### Secondary analyses

Exploratory analysis was performed to see if the same model could predict the individual components of 30-day MACE including all-cause mortality, cardiac death, and non-fatal MI.

## Abbreviations

AIC: Akaike information criteria
AUC: area under the ROC curve (also referred to as the c-statistic)
BNP: B-type natriuretic peptide concentration level (in pg/ml)
CI: confidence interval
df: degrees of freedom
GEE: generalized estimating equations
MACE: major adverse cardiac event
MELR: mixed effects logistic regression
MI: myocardial infarction
MPM threshold: indicator variable of BNP and NTproBNP thresholds determined by the minimum p value method
NTproBNP: N-terminal pro-B-type natriuretic peptide
OR: odds ratio
p: p value
RCRI: revised cardiac risk index
ROC: receiver operator characteristic curve
ROC threshold: indicator variable of BNP and NTproBNP thresholds determined by the ROC curve approach
RR: relative risk
SD: standard deviation
VIF: variance inflation factor
